# Genome-Wide Analysis in German Shepherd Dogs Reveals Association of a Locus on CFA 27 with Atopic Dermatitis

**DOI:** 10.1371/journal.pgen.1003475

**Published:** 2013-05-09

**Authors:** Katarina Tengvall, Marcin Kierczak, Kerstin Bergvall, Mia Olsson, Marcel Frankowiack, Fabiana H. G. Farias, Gerli Pielberg, Örjan Carlborg, Tosso Leeb, Göran Andersson, Lennart Hammarström, Åke Hedhammar, Kerstin Lindblad-Toh

**Affiliations:** 1 Science for Life Laboratory, Department of Medical Biochemistry and Microbiology, Uppsala University, Uppsala, Sweden; 2 Department of Clinical Sciences, Computational Genetics Section, Swedish University of Agricultural Sciences, Uppsala, Sweden; 3 Department of Clinical Sciences, Swedish University of Agricultural Sciences, Uppsala, Sweden; 4 Division of Clinical Immunology, Department of Laboratory Medicine, Karolinska Institute at Karolinska University Hospital Huddinge, Stockholm, Sweden; 5 Institute of Genetics, University of Bern, Bern, Switzerland; 6 Department of Animal Breeding and Genetics, Swedish University of Agricultural Sciences, Uppsala, Sweden; 7 Broad Institute of MIT and Harvard, Cambridge, Massachusetts, United States of America; Stanford University School of Medicine, United States of America

## Abstract

Humans and dogs are both affected by the allergic skin disease atopic dermatitis (AD), caused by an interaction between genetic and environmental factors. The German shepherd dog (GSD) is a high-risk breed for canine AD (CAD). In this study, we used a Swedish cohort of GSDs as a model for human AD. Serum IgA levels are known to be lower in GSDs compared to other breeds. We detected significantly lower IgA levels in the CAD cases compared to controls (p = 1.1×10^−5^) in our study population. We also detected a separation within the GSD cohort, where dogs could be grouped into two different subpopulations. Disease prevalence differed significantly between the subpopulations contributing to population stratification (λ = 1.3), which was successfully corrected for using a mixed model approach. A genome-wide association analysis of CAD was performed (*n*_cases_ = 91, *n*_controls_ = 88). IgA levels were included in the model, due to the high correlation between CAD and low IgA levels. In addition, we detected a correlation between IgA levels and the age at the time of sampling (corr = 0.42, p = 3.0×10^−9^), thus age was included in the model. A genome-wide significant association was detected on chromosome 27 (p_raw_ = 3.1×10^−7^, p_genome_ = 0.03). The total associated region was defined as a ∼1.5-Mb-long haplotype including eight genes. Through targeted re-sequencing and additional genotyping of a subset of identified SNPs, we defined 11 smaller haplotype blocks within the associated region. Two blocks showed the strongest association to CAD. The ∼209-kb region, defined by the two blocks, harbors only the *PKP2* gene, encoding Plakophilin 2 expressed in the desmosomes and important for skin structure. Our results may yield further insight into the genetics behind both canine and human AD.

## Author Summary

Humans and dogs are both affected by the allergic skin disease atopic dermatitis (AD), caused by an interaction between genetic and environmental factors. The German shepherd dog (GSD) is a high-risk breed for canine AD (CAD), also affected by low serum IgA levels. A Swedish cohort of GSDs was used as a model for human AD in this study. We performed a genome-wide association analysis where a region associated with CAD was identified. IgA levels were included in the model due to strong correlation with CAD. Also, age at sampling was included in the model due to correlation with IgA levels. The associated region, consisting of eight genes, was further fine-mapped with sequencing and additional genotyping. Haplotype association analysis from the fine-mapping data indicates association of the gene, *plakophilin 2* (*PKP2*), known to be important for skin structure. We detected a division of the GSD breed into two subpopulations where one is more prone to develop CAD and to have lower serum IgA levels compared with the other. Here, we present methods for performing genome-wide association analyses when the study population is complex and when the trait is affected by additional parameters. The *PKP2* gene found within the associated region became an interesting target for further study of its importance both in canine and human AD.

## Introduction

The domestic dog (*Canis familiaris*) has been bred for different purposes and characteristics for thousands of years [1]. The creation of modern dog breeds started around 200 years ago and was based on few founders and breeding strategies such as strong selection for certain traits, popular sires and inbreeding/backcrossing. This has led to enrichment of disease mutations in different breeds. The German shepherd dog (GSD) breed has an exceptionally high susceptibility to immunological diseases or immune-related disorders including skin as well as gastrointestinal problems. Inflammatory and immune-related diseases that have been reported with high incidence in GSDs are, for example exocrine pancreas insufficiency due to atrophy [2], [3], canine atopic dermatitis (CAD) [4], [5], anal furunculosis [6], [7] and disseminated aspergillosis [8]. A predisposition for food hypersensitivity and bacterial folliculitis [9] as well as low serum IgA levels [10]–[12] have also been reported in the GSD breed.

CAD is defined as an inflammatory and pruritic allergic skin disease caused by an interaction between genetic and environmental factors [13], [14]. The characteristic clinical features are most commonly associated with IgE antibodies directed towards environmental allergens [15]. In dogs, the allergic symptoms appear as eczematous skin but do not show the sequential development called atopic march (eczema in a child being often followed by asthma and allergic rhinitis in the adult patient) as described in humans [16], [17]. Clinical signs usually develop at a young age in both humans [16] and dogs. In dogs the disease onset is typically between six months and three years of age [18]. The initial signs of CAD can either be seasonal or non-seasonal, depending on the allergens involved. Face, ears, paws, extremities, ventrum and flex-zones are typically affected by pruritus and erythema [18] in a pattern similar to that observed in human AD [19]. To establish the diagnosis of CAD an extensive work-up is required [20], where conditions with similar clinical presentations must be ruled out. These include: scabies or other pruritic ectoparasite infestations, pruritic bacterial skin infections, Malassezia dermatitis, flea allergy dermatitis and, less commonly, cornification disorders and contact dermatitis. Cutaneous adverse food reactions (CAFR) can present similarly or contribute to clinical signs of CAD, but can be mediated by either hypersensitivity or non-immunological reactions. Thus, ideally the presence of CAFR should be evaluated before making the diagnosis. Also scabies could satisfy many of the inclusion criteria [21] and therefore has to be excluded as possible differential diagnosis. A positive allergen-specific IgE test (serology or intradermal test) is needed for final diagnosis and aids in defining offending allergens. In humans, mutations in the gene *filaggrin* (*FLG*) increase the risk of several complex diseases, including AD. Altogether 42% of AD-affected individuals carry *FLG* mutations, which is considerably higher than the carrier frequency of 10% observed in Europeans [22]. The aetiology of Filaggrin deficiency in AD is characterized by a cutaneous barrier defect, which enhances allergen penetration, bacterial colonisation and infection and cutaneous inflammation driven by type 2 helper T cells [23]. Filaggrin mutations are also known to cause asthma regardless of atopic phenotype [24] and ichtyosis vulgaris [25] in humans. Asthma-like symptoms are rarely reported in dogs: in a multi-centre study including ∼800 CAD dogs only 0.07% had any respiratory signs in the form of sneezing/rhinitis [17]. Different types of ichtyosis have been described in various breeds such as Golden retriever [26], Cavalier King Charles spaniel [27] and Soft Coated Wheaten terrier [28], however, to our knowledge, not in GSDs. Alopecia areata in humans has been correlated to *filaggrin* mutations and development of atopic dermatitis [29]. Canine models have previously been suggested for Alopecia areata [30], however this condition has not been reported in any dogs within our studied GSD population.

Immunoglobulin A (IgA) consists of two different forms, secretory IgA and serum IgA. In humans, serum concentrations of IgA are normally around 2–3 g/l, which makes it the second most prevalent antibody in serum after IgG [31]. IgA deficiency (IgAD) is the most common primary immunodeficiency in Caucasians with an estimated frequency of 1/600. IgA levels <0.07 g/l together with normal levels of IgG and IgM define IgAD in humans [32]. Compared to other dog breeds, very low IgA levels are known to be overrepresented in GSDs [33]–[37] Low serum IgA levels have also been reported in Shar-Pei [38] and Beagle [39]. Moreover, low levels of secretory-IgA in mucosa, tears [11], [40] and faecal extracts [41] have been reported in GSDs. Human studies show that children tend to have lower serum IgA levels than adults [42]. This is in concordance to the lower serum and secretory (tear) IgA levels being described in one year old or younger dogs compared to older dogs [43]. While increased incidence of upper respiratory tract infections, allergies and autoimmune diseases are observed in IgA-deficient human patients; more often humans show no symptoms at low levels of IgA [44]. Similarly, dogs with low IgA levels can either be asymptomatic or affected with recurrent upper respiratory infections and chronic dermatitis [39].

Due to the similarities between human patients and GSDs affected by AD and low IgA levels, we decided to study these two traits in a cohort of GSDs. Our aim was to detect loci associated with CAD and evaluate whether IgA levels in serum are correlated with the CAD phenotype in GSDs. We found a strong correlation between low serum IgA levels and CAD and could identify a genome-wide significant association of a locus with CAD using serum IgA levels and age at sampling as covariates. In addition to reaching our primary aim, we could also present characteristics specific to our sample cohort, including the detection of subpopulations with diverse predisposition of the studied phenotypes resulting in pronounced population stratification.

## Results

### Characterization of the sample cohort

**Figure 1:**
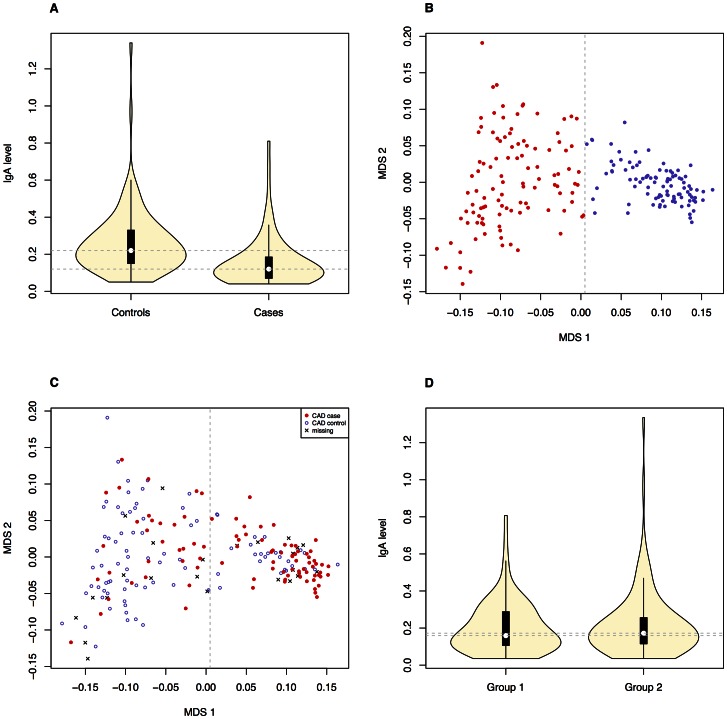
Correlation between the phenotypes and obvious population structure was detected in the GSD population. The difference in IgA levels (p_diff_ = 1.1×10^−5^, based on Welsh two sample t-test) in CAD cases and CAD controls (N_total_ = 184, before removing CAD controls with low IgA levels) is presented with boxplots (A). The GSD population is visualised with an MDS-plot displaying the formation of two subpopulations (B) with the uneven distribution of CAD cases and CAD controls (C). The distribution of IgA levels across the subpopulations is visualized with violin plots (D). Panels B-D include all dogs after QC (n = 203) and the dotted lines on the violin plots (A and D) correspond to the respective median values.

We investigated the diagnostic features CAD and low IgA levels, in a Swedish population of GSDs. The total number of dogs included in the study is presented in Table 1. When considering the CAD phenotype we first evaluated the relationship of the following parameters; CAD status, IgA levels and gender. 40.7% (n = 37) of the CAD cases had IgA-levels ≤0.10 g/l compared to 5.4% (n = 5) of the CAD controls. The IgA levels were significantly lower in CAD cases versus controls p = 1.1×10^−5^ (Figure 1A), mean IgA level in cases was 0.16 g/l and 0.26 g/l in controls (before excluding the 5 CAD controls with low IgA levels from the final association analysis, see *Materials and Methods*). We detected no gender bias in cases versus controls for CAD (p = 0.88). When considering whether IgA levels were related to age, we determined regression coefficient of 0.42 in all dogs together (p = 3.0×10^−9^), 0.37 in cases (p = 3.6×10^−4^) and 0.28 in controls (p = 8.5×10^−3^). We added the age at sampling as a covariate in the association analyses in order to remove any confounding effects of the IgA measurements' dependency of age.

**Table 1:** Individuals classified with CAD in the final analysis (before QC in brackets).

IgA levels	CAD cases	CAD controls	CAD missing	
≥0.20 g/l	21 (22)	57 (57)	0 (3)	
0.10–0.20 g/l	33 (35)	31 (31)	0 (0)	
IgA≤0.10 g/l	37 (37)	0 (5)	0 (1)	
**IgA missing**	0 (0)	0 (15)	0 (1)	
**Total**	91 (94)	88 (108)	0 (5)	179 (207)

### Genome-wide association studies (GWAS)

We performed genotyping of ∼170,000 SNP markers of the entire GSD cohort (n = 207). We excluded non-informative markers and markers with low call rate and 114,348 markers remained for the final analysis. We performed an association analysis of CAD using IgA levels and age at sampling as covariates.

### Extensive population stratification

The initial association analysis for CAD with IgA levels and age at sampling as covariates revealed that the GSD sample set was highly stratified with λ (genomic inflation factor) of λ_no correction_ = 1.3. The GSD population is clearly formed into two subpopulations (Figure 1B) defined using K-means clustering as described in *Materials and Methods*. The major cause of the high inflation factor, *i.e.* stratification, is the uneven distribution of cases and controls across the subpopulations visualized as a multi-dimensional scaling (MDS) plot (Figure 1C). In addition, the IgA levels followed a similar pattern, being unevenly distributed across the two subpopulations (Figure 1D). We found a pronounced difference in disease risk between subpopulations (p = 1.7×10^−6^, odds ratio OR = 4.4, CI_95_ = 2.3–8.8). The subpopulation counts are presented in Table 2.

**Table 2:** Summary of subpopulation statistics after QC.

	Subpopulation 1	Subpopulation 2
CAD controls	62	26
CAD cases	32	59
CAD status missing	13	11
IgA≥0.20 g/l	52	29
IgA 0.10–0.20 g/l	31	33
IgA≤0.10 g/l	18	25
IgA missing	6	9
Mean IgA level	0.23	0.19
**Total number of individuals**	**107**	**96**

**Figure 2:**
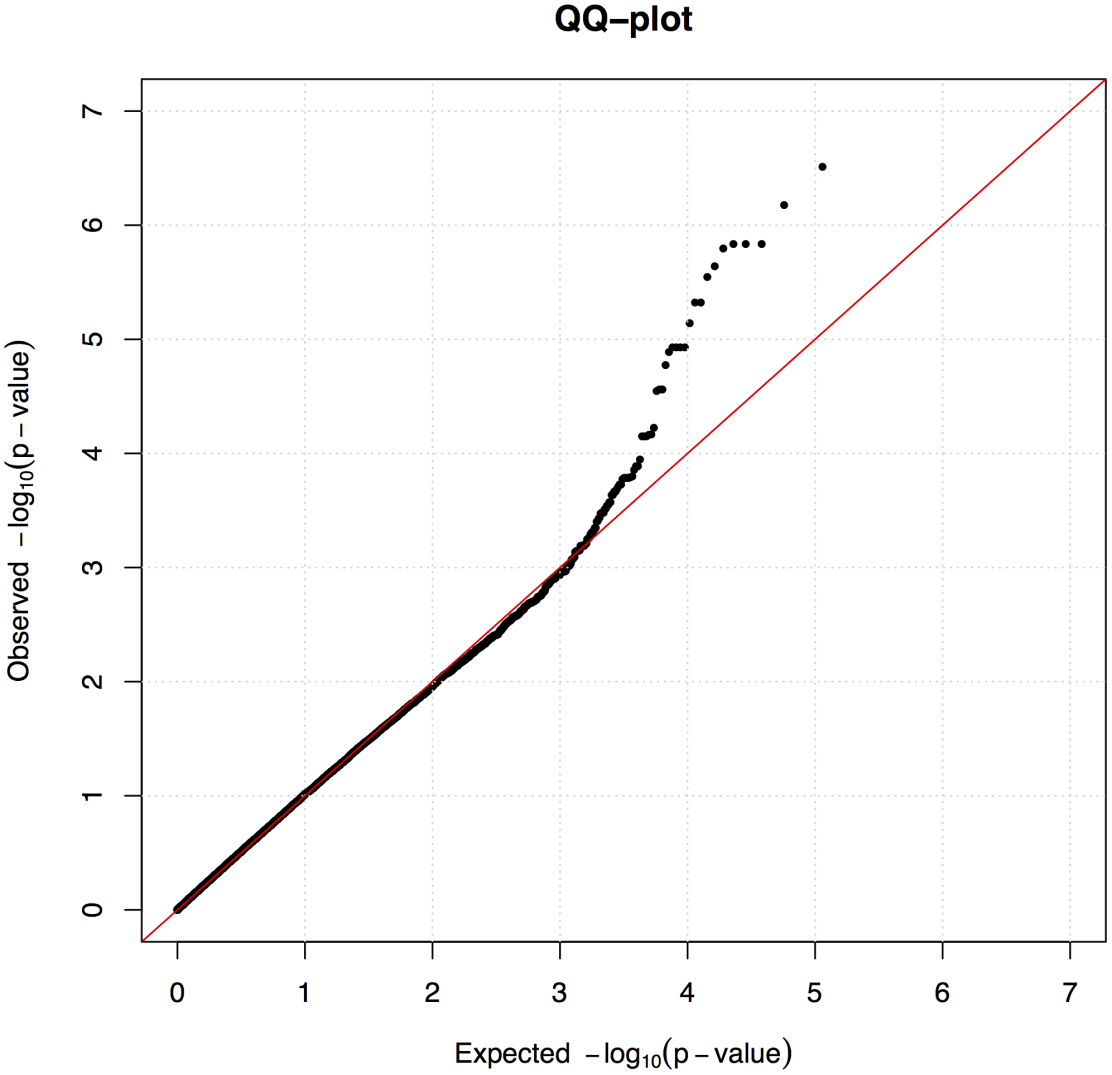
A mixed model corrected sufficiently for the population stratification. Quantile-Quantile (QQ) plot from the association analysis using the mixed model approach.

We used the mixed model approach to account for the observed population structure and cryptic relatedness between the individuals, which is common in dog breeds. After fitting the mixed model we observed no inflation (λ = 1.0) as presented in quantile-quantile (QQ) plot (Figure 2).

### A locus on chromosome 27 associated with CAD

**Figure 3:**
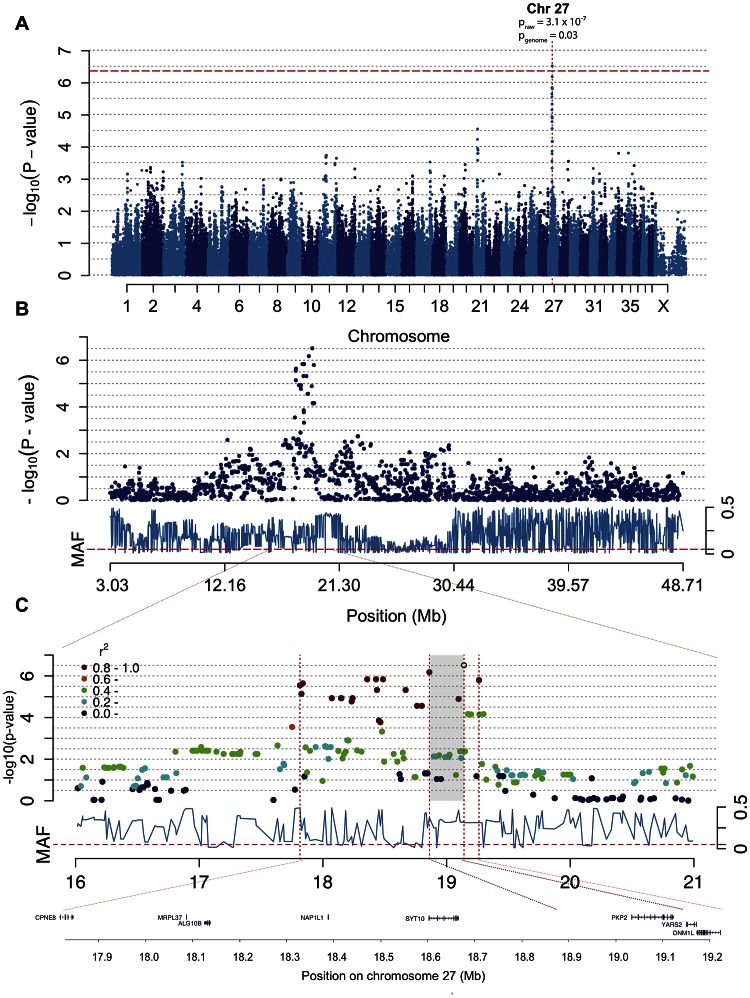
The associated region of ∼1.5 Mb on chromosome 27 includes the excellent candidate gene *PKP2*. Manhattan plot from the association analysis of CAD with IgA levels and age at sampling as covariates shows a significant association on chromosome 27. The red line represents Bonferroni-adjusted significance threshold of 0.05 (A). Chromosome 27 is displayed with association score for each SNP in dark blue and minor allele frequencies (MAF) in light blue below (B). The SNPs in high LD (r^2^≥0.8) with the top SNP are marked in red and the whole associated region is indicated by the outer dotted lines with the genes displayed below. The two top SNPs (shaded area) surround the *PKP2* gene (C).

In the association analysis of CAD we found a significant association to chromosome 27 where 19 SNPs between 17,814,493–19,262,027 (CanFam 2.0) showed association p<2.8×10^−5^. The top two SNPs are located at canine chromosome 27 (CFA 27): 19,140,837 bp (p_raw_ = 3.1×10^−7^ and p_genome_ = 0.03) and 18,861,228 bp (p_raw_ = 6.7×10^−7^and p_genome_ = 0.07) (Figure 3A–3C). To define the associated haplotype we performed clumping using r^2^ = 0.8, and identified a 21 SNP haplotype spanning from 17,814,493 to 19,262,027. This haplotype region contains eight genes (*CPNE8, MRPC37, ALG10B, NAP1L1, SYT10, PKP2, YARS2* and *DNM1L*) where the two top SNPs surround the *PKP2* gene as indicated in Figure 3C. The haplotype corresponds to the region identified by the 19 associated SNPs and covers a region of ∼1.5 Mb. The haplotype region shows a mosaic pattern of association typical for purebred dogs [45], thus it is not possible from this data to define a shorter associated haplotype.

Using Haploview we detected lower association to CAD when considering the ∼1.5 Mb haplotype compared to the single top SNPs (p_haplotype_ = 2.6×10^−5^). The observed minor allele frequency (MAF) of the top SNP (CFA 27: 19,140,837 bp) was 0.29 across all samples, and 0.40 and 0.16 in cases and controls, respectively. The minor allele (G) conferred an OR = 1.28 for CAD. We observed a two-fold difference in MAF between the two detected subpopulations (MAF_subpopulation 1_ = 0.40, MAF_subpopulation 2_ = 0.20).

### Targeted re-sequencing of the associated locus on CFA 27

**Figure 4:**
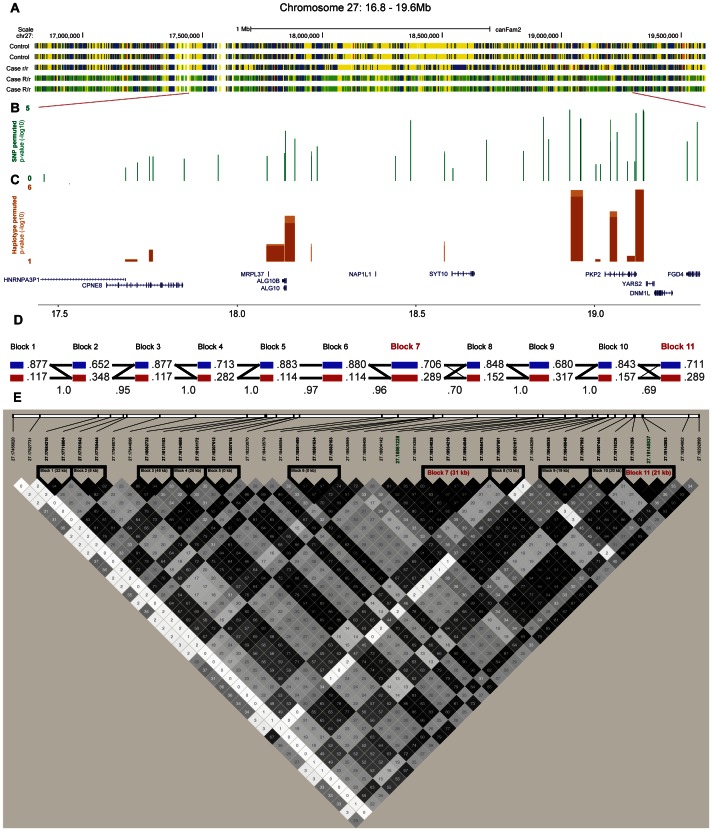
Fine-mapping of the chromosome 27 locus confirms the association with CAD and further pinpoints the region around the *PKP2* gene. Targeted re-sequencing data is shown for five dogs; two controls (homozygous for the control allele R/R), one case (homozygous for the risk allele r/r) and two cases (carriers of the risk allele R/r) in panel A. Yellow markings show SNPs that are homozygous for the reference allele, blue are homozygous for the non reference allele, green are heterozygous and red are SNPs in conserved elements. The association (p-value after 1,000,000 permutations) of the genotyped SNPs (n = 42) and haplotypes (n = 11) are presented in panel B and C, respectively. The haplotype blocks, the correlation between blocks (thin lines >1.0%, thick lines >10 %) and frequencies of alleles in the genotyped GSD population are presented in D. Haplotype blocks were defined by r^2^≥0.9, r^2^ values are presented in each square where black represent the highest value and white the lowest (E). Each SNP, named after its position in the genome, are shown above, where the two top GWAS SNPs are marked green and the top associated haplotype blocks (7 and 11) are marked red.

We performed targeted re-sequencing (Roche NimbleGen sequence capture array) of the locus on CFA 27 spanning 16.8–19.6 Mb (CanFam 2.0) *i.e.* including the associated haplotype located at ∼17.8–19.3 Mb. In total, three dogs homozygous for the control haplotype, one dog homozygous for the case haplotype and three dogs heterozygous for the case and control haplotypes were sequenced (Figure 4A). In total, 2,587 SNPs of all the identified SNPs (n = 8,765) followed the case and control haplotype pattern (see *Materials and Methods*). We used SEQScoring [46], (see *Materials and Methods*) to prioritize potentially causal variants. As expected, the majority of the SNPs detected to correlate with the case/control haplotypes (86%) were located within the associated (17.8–19.3 Mb) region. No structural variants were detected. In total, 54 SNPs were included on an iPLEX array for further genotyping in the same cohort used for the GWAS. These SNPs were concordant with the risk haplotype and considered functional candidates based on their location in conserved elements or in genes. In addition the top GWAS SNPs were included. For the final analysis, 42 SNPs and 84 controls and 91 cases remained after quality control (see *Materials and Methods*). Using Haploview, we defined haplotypes based on r^2^≥0.9 between neighbouring SNPs. The risk alleles of block 11 and 7 (GCCA and AGG, respectively) had a frequency of 40.1% in the cases versus 16.7% in the controls (p_raw_ = 1.3×10^−6^, p_1,000,000perm_ = 4.0×10^−6^). The common control allele TTT of block 11 had the same p-value as the risk allele and a frequency of 83.3 % in controls versus 59.9% in cases. Considering single SNPs; the top associated were the risk alleles of 18,934,038 bp and 18,934,219 bp (part of block 7), and 19,140,837 bp (part of block 11 and also the top GWAS SNP). They had the same frequency as the risk alleles of the corresponding haplotypes and were associated to the same extent (p_raw_ = 1.3×10^−6^) but with a slightly less significant p-value after permutations (p_1,000,000perm_ = 3.1×10^−5^) due to the larger number of SNPs compared to haplotypes. See the association analysis results of haplotypes and SNPs in Table 3 and Table 4, respectively (see also Tables S1 and S2). The association of SNPs and haplotypes (p-value after 1,000,000 permutations) as well as the defined haplotypes and the LD plot are visualized in Figure 4B–4E. These results indicate that the region; 18,934,038 – 19,142,893 Mb harbours the causative mutation predisposing for CAD in the studied GSD population. This is in concordance with the genome-wide association results where the top associated SNP is located at 19,140,837 bp. Only one gene, *PKP2*, falls within the top region (defined by block 7–11). The *PKP2* gene, encoding the protein Plakophilin 2, a central component of desmosomes [47], is an excellent candidate gene for CAD.

**Table 3:** Top 10 haplotype alleles from the association analysis of fine-mapping data.

		Frequency			
Block	Allele	Case	Control	p-value	p_1,000,000 permutations_
7	**GCCA**	**0.401**	**0.167**	1.3×10^−6^	4.0×10^−6^
11	**AGG**	**0.401**	**0.167**	1.3×10^−6^	4.0×10^−6^
11	TTT	0.599	0.833	1.3×10^−6^	4.0×10^−6^
7	TAAC	0.599	0.821	5.0×10^−6^	2.8×10^−5^
9	TAT	0.418	0.208	2.7×10^−5^	1.0×10^−4^
4	AA	0.378	0.179	3.7×10^−5^	2.0×10^−4^
9	CGC	0.582	0.786	4.6×10^−5^	3.0×10^−4^
4	CG	0.622	0.810	1.0×10^−4^	9.0×10^−4^
6	TTC	0.824	0.940	8.0×10^−4^	0.0060
3	AT	0.170	0.060	0.0013	0.0079

**Table 4:** Top 15 SNP alleles from the association analysis of fine-mapping data.

		Frequency			
Position	Allele	Case	Control	p-value	p_1,000,000 permutations_
18934038^7^	**G**	**0.401**	**0.167**	1.3×10^−6^	3.1×10^−5^
18934219^7^	**C**	**0.401**	**0.167**	1.3×10^−6^	3.1×10^−5^
19140837^11^	**G**	**0.401**	**0.167**	1.3×10^−6^	3.1×10^−5^
19142893^11^	G	0.400	0.167	1.5×10^−6^	3.2×10^−5^
19121205^11^	A	0.401	0.169	1.8×10^−6^	4.4×10^−5^
18861228	A	0.390	0.167	3.5×10^−6^	6.9×10^−5^
18964049^7^	C	0.401	0.179	5.0×10^−6^	9.4×10^−5^
18965475^7^	A	0.401	0.179	5.0×10^−6^	9.4×10^−5^
18486594	A	0.390	0.173	6.8×10^−6^	1.0×10^−4^
19292898	T	0.401	0.185	9.4×10^−6^	2.0×10^−4^
19048938	T	0.417	0.208	3.0×10^−5^	5.0×10^−4^
19049048	A	0.417	0.208	3.0×10^−5^	5.0×10^−4^
18134508	A	0.378	0.179	3.7×10^−5^	7.0×10^−4^
19067992	T	0.418	0.214	4.6×10^−5^	8.0×10^−4^
18161172	A	0.378	0.190	1.0×10^−4^	0.0020

7 *SNPs part of block 7*,

11 *SNPs part of block 11*.

## Discussion

### Genome-wide association of CAD

We detected a significant difference in IgA levels in CAD cases compared to CAD controls (Figure 1A), using a mixed-model approach. This suggests a functional role of IgA in the aetiology of CAD. The overall low IgA levels seen in the GSD breed might contribute to its predisposition for CAD: among the CAD cases 40.7% had low IgA-levels compared to only 5.4% of the CAD controls.

The associated haplotype on chromosome 27 from the genome-wide association analysis of CAD includes eight genes; *CPNE8, MRPC37, ALG10B, NAP1L1, SYT10, PKP2, YARS2* and *DNM1L*.

### Subpopulations in the GSD breed

The first German sheepdogs were exhibited in 1882 at a dog show in Hannover, Germany. These dogs were the ancestors to what became the German shepherd dog (GSD) breed formed in 1899. The way breeding has been performed led to a split into two variants in the end of the 1970s [48].

The Swedish GSD population used in this study was highly stratified primarily due to the formation of two subpopulations. We found a significant difference between subpopulations regarding both phenotypes in the study (IgA levels and CAD) where subpopulation 2 harbours more CAD cases and dogs with low IgA levels than subpopulation 1. When comparing the merits of the dogs included in the CAD association analysis, we noted that GSDs in subpopulation 1 were more often of working type compared to subpopulation 2. Moreover, fewer dogs in subpopulation 1 had documented show results compared to subpopulation 2. Thus, the risk of CAD and low IgA levels seems lower in the GSD population bred for working capacity.

The stratification was successfully corrected for by using the mixed model approach within the GenABEL software. Not only does it correct for the formation of two clusters and the uneven distribution of cases and controls across the clusters, but also for cryptic relatedness typical for dog breeds. Despite the identified subpopulations, there is no apparent discontinuity between them in terms of gene flow (Figure 1B). Therefore, a mixed model approach was sufficient to remove the effect of stratification. Simpler approaches, such as genomic control or PCA-based corrections, were not capable of correcting the observed stratification (data not shown). In addition, we used IgA levels and age at sampling as covariates in order to account for their effect on the observed phenotypes.

### Candidate mutation detection and validation genotyping of the CFA 27–associated region

The sequencing data generated in the 2.8 Mb region on CFA 27 verified the ∼1.5 Mb long associated haplotype showing 86% of the 2,587 SNPs following the case and control haplotype pattern located at ∼17.8–19.3 Mb. Based on further genotyping of 42 SNPs within the region there is clear indication that the region 18.94–19.14 Mb, based on both haplotypes and single SNPs, harbours the mutation predisposing for CAD in GSDs. By performing targeted re-sequencing of the associated region we attempted to identify all variants concordant with the phenotype and then evaluate their potential as risk variants. Here we identified two haplotypes with multiple SNPs with equally strong association and a potential for function. While one or several of these variants may be the causative variant, it is also possible that actual mutation may have been missed in the targeted re-sequencing process or in the genotyping process as several SNPs failed genotyping for technical reasons. Furthermore, our ability to predict functionality is not comprehensive as functional variants may be located in non-conserved elements or in complicated regions with low sequence coverage. The actual functional variant may also be an indel or CNV not identified in this analysis. Further analysis should reveal the exact causative mutation.

The gene *PKP2*, encoding Plakophilin 2, is the only gene located within the associated 200 kb region. Plakophilin proteins are localized in the desmosomal plaque and cell nucleus and participate in linking cadherins to intermediate filaments in the cytoskeleton [49]. Plakophilin 2 takes part in pathways that drive actin reorganization and regulation of desmoplakin-intermediate filament interactions required for normal desmosome assembly [50]. Changes in the corneodesmosomes (modified desmosomes in the epidermis) degradation process influence the thickness of the stratum corneum and surface of the skin and abnormal corneodesmosome degradation has been found in common skin diseases including atopic dermatitis [51]. A recent small study in dogs showed statistically significant altered mRNA expression of *PKP2* between atopic and healthy skin (20 cases and 17 controls of various breeds and mongrels). In addition, the expression correlated with clinical severity in atopic skin [52]. Defective permeability barrier function enables enhanced infiltration of environmental allergens into the skin, which in turn triggers immunological reactions and inflammation. [53]. Based on the increasing evidence of the skin barrier being a crucial component in the development of human and canine atopic dermatitis [54]–[55], *PKP2* serves as an excellent candidate gene. Furthermore, Filaggrin is known as a filament-aggregating protein and it is important for the formation of the stratum corneum, the outermost layer of epidermis [25]. Since the desmosome is one of the best characterized components of the stratum corneum [56] the importance of Filaggrin and Plakophilin 2 for skin structure in the aetiology of AD may be very similar.

### Conclusions

Further studies are necessary to conclusively define how CAD and low IgA levels are correlated. Low IgA levels may also affect other immune-related diseases that occur in the GSD breed. The results presented here set a starting point for further studies of susceptibility to immune diseases within the GSD breed. Even more importantly a novel gene, *PKP2*, is indicated to be involved in the development of CAD in GSDs. This may be of significance also in other dog breeds and in human AD.

## Materials and Methods

### Sampling and ethics statement

We collected blood samples (EDTA for DNA extraction and serum for IgA measurements) from 207 German shepherd pet dogs in collaboration with veterinary clinics throughout Sweden. Owner consent was collected for each dog. The majority of dogs included in the study were registered in the Swedish Kennel club (180 out of 207). We conformed the sampling to the approval of the Swedish Animal Ethical Committee (no. C62/10) and the Swedish Animal Welfare Agency (no. 31-1711/10).

### Samples

We extracted genomic DNA from the EDTA blood samples using the Qiagen mini- and/or midiprep extraction kit (Qiagen, Hilden, Germany). DNA samples were diluted in de-ionized water and stored at −20°C. Serum was separated from the red blood cells by centrifugation and then stored at −20/−80°C.

### CAD phenotype characterization

The CAD cases were dogs of all ages with positive reactions on allergen-specific IgE test (intradermal test or IgE serology test), either with or without concurrent cutaneous adverse food reactions (CAFR). Clinical diagnoses were established by first ruling out other causes of pruritus such as ectoparasite infestation, staphylococcal pyoderma and *Malassezia* dermatitis. A hypoallergenic dietary trial (at least 6–8 weeks followed by a challenge period) was then conducted in order to evaluate the potential contribution of CAFR. Atopic reactions were concluded if the dog was not adequately controlled on hypoallergenic diet and had positive reactions on intradermal allergy tests (skin prick test) or IgE serology tests.

All CAD controls were over five years of age and had never suffered from pruritus, repeated ear inflammations or skin lesions compatible with CAD, neither prior to nor at the time of sampling. The age cut-off for CAD controls was set at five since affected dogs rarely debut at ages older than three years of age [17], [18]. The information was based on either owner questionnaire and/or clinical examination. In addition, we excluded dogs with low IgA levels (IgA≤0.10 g/l) as CAD controls.

### Measurements of serum IgA

We measured serum IgA concentrations with enzyme-linked immunosorbent assay (ELISA) using polyclonal goat anti-dog IgA antibodies (AbD Serotec, Oxford, UK), polyclonal mouse anti-dog IgA antibodies (AbD Serotec) and polyclonal, AP-conjugated goat anti-mouse IgG (Jackson Immunoresearch, West Grove, PA). All antibodies were diluted 1∶2,000 in PBS and the serum samples were diluted 1∶25,000; 1∶50,000 and 1∶100,000 in PBS. All samples were measured at least twice. The coefficient of variation (CV) was calculated. Samples with a CV value ≥15% were measured again. Before the average concentration was calculated, potentially outlying concentrations were excluded. With a maximal variation of 15% the reproducibility of our measurements are in the lower range of ELISA measurements which can be as high as 25%.

Dogs with serum IgA levels ≤0.10 g/l were considered to be IgA-deficient and thus not deemed appropriate controls for CAD. All the dogs were sampled at the age of more than one year except for one individual that was 11 months and 13 days at the time of sampling.

### Statistical analyses of traits and covariates

We examined the relationships between measured phenotypes and other possible covariates. We used Fisher's exact test for count data to determine whether CAD-gender relationships were significant. Similarly we used the Welch two-sample t-test for determining the CAD-IgA levels relationship. We used the same approaches to check if there were any significant differences in CAD status or IgA levels between subpopulations.

As IgA levels can vary with age, we fitted a linear model to determine the age effect on the IgA levels, and used Pearson's correlation coefficient to measure the strength of the relationship. We considered CAD cases and controls separately and together. The age at the time of sampling was defined at 0.1-year resolution for most individuals and estimated at a year resolution for 10 dogs (n_controls_ = 7, n_cases_ = 3).

### SNP genotyping and quality control

The initial data set consisted of 207 individuals genotyped using the Illumina 170K CanineHD BeadChip (Illumina, San Diego, CA). Summary of individuals in each trait class is presented in Table 1, before and after quality control (QC).

Prior to principal GWAS, we performed iterative QC to remove poorly genotyped and noisy data. Out of the initial number of 174,376 SNP markers, we excluded 55,399 (31.77%) non-informative markers (minor allele frequency below 1%), 2,537 (1.45%) due to call rate below 0.95 and 2,722 (1.56%) markers due to the departure from Hardy-Weinberg equilibrium (first p<1×10^−8^ and then FDR<0.2 in CAD controls only). In total, 114,348 markers (65.57%) were included in both analyses.

Considering the entire dataset consisting of 207 individuals, we excluded two individuals due to exceptionally high identity-by-state, IBS>0.95 (the one with lowest call rate was excluded in each pair - all were CAD cases) and two apparent outliers on the multidimensional scaling (MDS) plot resulting in 203 individuals passing QC. After QC, 25 individuals in total were excluded from the association analysis; five were missing CAD status, five CAD controls had low IgA levels and 15 CAD controls were missing IgA levels (Table 1).

The initial association (with IgA levels and age at sampling as covariates) indicated population stratification (λ = 1.3, λse = 1.5×10^−3^). Hence, we decided to perform a closer examination of the genetic structure of our GSD population by computing autosomal genomic kinship matrix and performing standard K-means clustering. In order to determine the number of clusters (subpopulations), we performed a number of K-means clustering with K = {1,2, …, 10}. At each iteration, we were computed and stored the sum of within-cluster sums of squares (ΣWCSS). Subsequently, we used the so-called scree test by plotting ΣWCSS vs. K and choosing the number of clusters (K = 2) corresponding to the first inflection point (for details see: [57]). The clusters define our subpopulations.

Using MDS, we present visualisation of the genomic-kinship matrix and subpopulations in Figure 1B–1C, and subpopulation statistics are shown in Table 2.

### Genome-wide association analysis

We performed association analysis of CAD (91 cases and 88 controls) with IgA levels and age at sampling as covariates. We used the GenABEL package ver. 1.7-0 [58], a part of R statistical suite/software, ver. 2.14.2 [59] for the genome-wide association analyses. We used the mixed model approach for all the final analysis presented in this paper. Mixed models were fitted using polygenic_hglm function from the hglm package ver. 1.2–2 [60]. All parameters used for functional calls are discussed in the paragraphs describing particular steps of the previous sections. We considered p-values below 0.05 (p_raw_) as significant and after 100,000 permutations as genome-wide significant p-values (p_genome_).

For haplotype definitions we performed LD-clumping (settings; r^2^ = 0.8, p1 = 0.0001, p2 = 0.001, distance d = 3 Mb) using our own R implementation of the algorithm described in the PLINK documentation (PLINK v1.07, [61]) and Haploview 4.2 (version 1.0).

### Targeted re-sequencing

We selected five individuals for targeted re-sequencing of the CFA 27 locus. A single case was homozygous for the risk haplotype and two were heterozygous, whereas two controls lacked the risk haplotype. Targeted capture of in total 6.5 Mb out of which 2.8 Mb spanning CFA 27:16.8–19.6 Mb (CanFam 2.0) including the ∼1.5 Mb associated haplotype, was performed using a 385K custom-designed sequence capture array from Roche NimbleGen, WI. Hybridization library preparation was performed as described by Olsson et al. [62]. Captured enriched libraries were sequenced with a read length of 100 bp (paired-end reads), using HiSeq 2000 (Illumina sequencing technology). Sequencing was performed by the SNP&SEQ Technology Platform at SciLifeLab Uppsala. Obtained reads were mapped to CanFam 2.0 [45] using Burrows-Wheeler Aligner (BWA) [63]. The Genome Analysis Toolkit (GATK) (http://www.broadinstitute.org/gatk, all web resources used in this study are also summarized in Text S1) was used for base quality recalibration and local realignment and the tool picard (hosted by SAMtools [64]) for removing PCR duplicates. For variant calling SAMtools/0.1.18 was applied using mpileup format and bcftools. Maximum read depth to call a SNP (-D) was set to 300 and the function -C50 was applied to reduce the effect of reads with excessive mismatches (http://samtools.sourceforge.net). Mean coverage in the five analyzed individuals was 66.9 reads and mean share of positions covered by at least 10 reads was 87% (Table S3). We searched for structural variants by performing depth of coverage analyses using average coverage for controls as a reference. Coverage was calculated using every 20-th position in the raw pileup files and then normalized for every individual. Next, the coverage was averaged within a 100 positions-wide window separately for controls and cases. The average cases/controls ratio was then computed and used as indicator of a copy-number variation. In regions with reduced (<−1.0) or elevated (>1.0) relative coverage, we additionally examined the length of inferred insert size using the integrative genomics viewer (IGV) [65].

We used SEQScoring [46] (http://www.seqscoring.net) to score the SNPs by conservation and haplotype pattern; and the integrative genomics viewer (IGV) was used for manual visualization of SNPs, individual coverage and indels. In total, 8,765 SNPs were identified in the chromosome 27 region. Out of these, 2,587 SNPs followed the pattern of the case and control haplotypes defined by the top GWAS SNPs. The pattern was based on three dogs homozygous for the control haplotype, one dog homozygous for the case haplotype and three dogs carrying the case and control haplotype (*i.e.* carriers of the case haplotype). Out of the 2,587 SNPs only 46 SNPs were located within conserved elements (+/−5 bp) scored by SEQscoring according to SiPhy constraint elements detected by the alignment of 29 eutherian mammals [66]. We picked out 60 SNPs for designing a genotyping array. The selection was based on the following criteria; 40 SNPs out of the 46 SNPs stated above (SNPs too close to each other and located in repeated sequences were excluded), SNPs from the genome-wide array for comparison, manually picked SNPs within the *PKP2* gene (not conserved) and SNPs in gaps in order to cover the entire associated region. Out of these, 54 SNPs were successfully pooled for additional genotyping in all dogs.

### Genotyping of fine-mapping SNPs

The 54 SNPs were genotyped using iPLEX Sequenom MassARRAY platform (http://www.sequenom.com/iplex) in 185 GSD dogs. After analyzing the quality of the SNP genotyping 12 SNPs were excluded due to bad calling; nine due to heterozygotes were incorrectly called as homozygous and two due to one of the homozygous genotypes was falsely called as heterozygous and one due to MAF = 0. In total, 42 SNPs remained for the analysis. For the association analysis of the genotyped SNPs and for defining haplotypes we used Haploview 4.2 (version 1.0). In total, 84 controls and 91 cases were included in the analysis – the same set as in the genome-wide association analysis of CAD except for four excluded controls (two were not included due to missing DNA and two were excluded due to low call rate = 48%).

## Supporting Information

Table S1 Top 22 haplotype alleles from the association analysis of fine-mapping data.
(PDF)

Table S2 Top 42 SNP alleles from the association analysis of fine-mapping data.
(PDF)

Table S3 Positions covered by at least 5 and by at least 10 reads.
(PDF)

Text S1 Web resources.
(PDF)
