# Correction: Genome-Wide Analysis in German Shepherd Dogs Reveals Association of a Locus on CFA 27 with Atopic Dermatitis

**DOI:** 10.1371/annotation/fd6f4425-3d84-4017-a012-a5df6ddee13a

**Published:** 2013-05-16

**Authors:** Katarina Tengvall, Marcin Kierczak, Kerstin Bergvall, Mia Olsson, Marcel Frankowiack, Fabiana H. G. Farias, Gerli Pielberg, Örjan Carlborg, Tosso Leeb, Göran Andersson, Lennart Hammarström, Åke Hedhammar, Kerstin Lindblad-Toh

The following information was missing from the Competing Interests section: "This work has been filed as a US provisional patent application."

