# Correction: Genome-Wide Analysis in German Shepherd Dogs Reveals Association of a Locus on CFA 27 with Atopic Dermatitis

**DOI:** 10.1371/journal.pgen.1005740

**Published:** 2015-12-11

**Authors:** Katarina Tengvall, Marcin Kierczak, Kerstin Bergvall, Mia Olsson, Marcel Frankowiack, Fabiana H. G. Farias, Gerli Pielberg, Örjan Carlborg, Tosso Leeb, Göran Andersson, Lennart Hammarström, Åke Hedhammar, Kerstin Lindblad-Toh

The following information is missing from the Funding section: KL-T was also supported by the European Research Council (ERC).
