# Supplementary material for: Genome-Wide Analysis in German Shepherd Dogs Reveals Association of a Locus on CFA 27 with Atopic Dermatitis
Source: PLoS Genet. 2013 May 9;9(5):e1003475. doi: 10.1371/journal.pgen.1003475 (PMC3649999; doi:10.1371/journal.pgen.1003475)
Supplement: Table S1 — Top 22 haplotype alleles from the association analysis of fine-mapping data. (PDF) [file pgen.1003475.s001.pdf]

		Frequency			
**Block**	**Allele**	**Case**	**Control**	**p-value**	**$p_{1,000,000\ permutations}$**
7	**GCCA**	**0.401**	**0.167**	1.3×10^{-6}	4.0×10^{-6}
11	**AGG**	**0.401**	**0.167**	1.3×10^{-6}	4.0×10^{-6}
11	TTT	0.599	0.833	1.3×10^{-6}	4.0×10^{-6}
7	TAAC	0.599	0.821	5.0×10^{-6}	2.8×10^{-5}
9	TAT	0.418	0.208	2.7×10^{-5}	1.0×10^{-4}
4	AA	0.378	0.179	3.7×10^{-5}	2.0×10^{-4}
9	CGC	0.582	0.786	4.6×10^{-5}	3.0×10^{-4}
4	CG	0.622	0.810	1.0×10^{-4}	9.0×10^{-4}
6	TTC	0.824	0.940	8.0×10^{-4}	0.0060
3	AT	0.170	0.060	0.0013	0.0079
5	TG	0.830	0.940	0.0013	0.0079
3	GC	0.824	0.935	0.0017	0.0113
6	CCA	0.165	0.060	0.0020	0.0129
5	AA	0.165	0.060	0.0020	0.0133
2	CT	0.578	0.732	0.0025	0.0163
2	AA	0.422	0.268	0.0025	0.0163
10	AC	0.791	0.899	0.0057	0.0379
10	GT	0.209	0.101	0.0057	0.0379
8	AT	0.8	0.899	0.0104	0.0591
8	GC	0.2	0.101	0.0104	0.0591
1	AA	0.159	0.071	0.0106	0.0612
1	GG	0.835	0.923	0.0128	0.0719
