# Supplementary material for: Genome-Wide Analysis in German Shepherd Dogs Reveals Association of a Locus on CFA 27 with Atopic Dermatitis
Source: PLoS Genet. 2013 May 9;9(5):e1003475. doi: 10.1371/journal.pgen.1003475 (PMC3649999; doi:10.1371/journal.pgen.1003475)
Supplement: Table S2 — Top 42 SNP alleles from the association analysis of fine-mapping data. (PDF) [file pgen.1003475.s002.pdf]

		Frequency			
Position	Allele	Case	Control	p-value	$p_{1,000,000\ permutations}$
18934038[7]	**G**	**0.401**	**0.167**	1.3×10^{-6}	3.1×10^{-5}
18934219[7]	**C**	**0.401**	**0.167**	1.3×10^{-6}	3.1×10^{-5}
19140837[11]	**G**	**0.401**	**0.167**	1.3×10^{-6}	3.1×10^{-5}
19142893[11]	G	0.400	0.167	1.5×10^{-6}	3.2×10^{-5}
19121205[11]	A	0.401	0.169	1.8×10^{-6}	4.4×10^{-5}
18861228	A	0.390	0.167	3.5×10^{-6}	6.9×10^{-5}
18964049[7]	C	0.401	0.179	5.0×10^{-6}	9.4×10^{-5}
18965475[7]	A	0.401	0.179	5.0×10^{-6}	9.4×10^{-5}
18486594	A	0.390	0.173	6.8×10^{-6}	1.0×10^{-4}
19292898	T	0.401	0.185	9.4×10^{-6}	2.0×10^{-4}
19048938	T	0.417	0.208	3.0×10^{-5}	5.0×10^{-4}
19049048	A	0.417	0.208	3.0×10^{-5}	5.0×10^{-4}
18134508	A	0.378	0.179	3.7×10^{-5}	7.0×10^{-4}
19067992	T	0.418	0.214	4.6×10^{-5}	8.0×10^{-4}
18161172	A	0.378	0.190	1.0×10^{-4}	0.0020
18874358	A	0.404	0.214	1.0×10^{-4}	0.0024
18699406	G	0.187	0.054	1.0×10^{-4}	0.0026
19264902	T	0.192	0.060	2.0×10^{-4}	0.0036
18223070	G	0.374	0.202	4.0×10^{-4}	0.0067
18804142	G	0.409	0.241	0.0010	0.0147
18582103	A	0.172	0.060	0.0011	0.0164
18131103	T	0.170	0.060	0.0013	0.0187
18207512	A	0.170	0.060	0.0013	0.0187
18581634	C	0.170	0.060	0.0013	0.0187
17944696	T	0.156	0.054	0.0020	0.0278
18082732	A	0.172	0.065	0.0023	0.0308
18443579	T	0.172	0.065	0.0023	0.0308
17751542	A	0.422	0.268	0.0025	0.0333
17760444	A	0.422	0.268	0.0025	0.0333
18581490	C	0.165	0.061	0.0026	0.0336
17848875	C	0.390	0.244	0.0034	0.0436
18207618	A	0.157	0.060	0.0036	0.0464
19097445	G	0.209	0.101	0.0057	0.0729
19118236	T	0.209	0.101	0.0057	0.0729
17716804	A	0.165	0.071	0.0072	0.0868
19007501	G	0.200	0.101	0.0104	0.1284
19021017	C	0.200	0.101	0.0104	0.1284
19048269	A	0.209	0.113	0.0154	0.1682
17684210	A	0.159	0.077	0.0184	0.1960
18605999	G	0.198	0.107	0.0190	0.1998
17456020	C	0.208	0.131	0.0573	0.4301
17527731	G	0.005	0.000	0.3360	0.9719

[7]*SNPs part of block 7,* [11]*SNPs part of block 11*
