# Supplementary material for: Genome-Wide Analysis in German Shepherd Dogs Reveals Association of a Locus on CFA 27 with Atopic Dermatitis
Source: PLoS Genet. 2013 May 9;9(5):e1003475. doi: 10.1371/journal.pgen.1003475 (PMC3649999; doi:10.1371/journal.pgen.1003475)
Supplement: Table S3 — Positions covered by at least 5 and by at least 10 reads. (PDF) [file pgen.1003475.s003.pdf]

	5X	10X	Mean
T1	0.967	0.945	86.402
T2	0.819	0.619	16.411
T3	0.946	0.909	47.102
T4	0.972	0.959	109.874
T5	0.959	0.933	74.747
**Sum**	**4.663**	**4.362**	**334.536**
**Mean**	**0.933**	**0.872**	**66.907**
