# Supplementary material for: Genome-Wide Analysis in German Shepherd Dogs Reveals Association of a Locus on CFA 27 with Atopic Dermatitis
Source: PLoS Genet. 2013 May 9;9(5):e1003475. doi: 10.1371/journal.pgen.1003475 (PMC3649999; doi:10.1371/journal.pgen.1003475)
Supplement: Text S1 — Web resources. (PDF) [file pgen.1003475.s004.pdf]

http://www.broadinstitute.org/gatk

http://www.seqscoring.net

http://www.sequenom.com/iplex

http://samtools.sourceforge.net
